# Different modes of state transitions determine pattern in the Phosphatidylinositide-Actin system

**DOI:** 10.1186/1471-2121-12-42

**Published:** 2011-10-07

**Authors:** Günther Gerisch, Mary Ecke, Dirk Wischnewski, Britta Schroth-Diez

**Affiliations:** 1 Max Planck Institute of Biochemistry, Am Klopferspitz 18, 82152 Martinsried, Germany; 2 Max Planck Institute of Molecular Cell Biology and Genetics, Pfotenhauerstrasse 108, 01307 Dresden, Germany

## Abstract

**Background:**

In a motile polarized cell the actin system is differentiated to allow protrusion at the front and retraction at the tail. This differentiation is linked to the phosphoinositide pattern in the plasma membrane. In the highly motile *Dictyostelium *cells studied here, the front is dominated by PI3-kinases producing PI(3,4,5)tris-phosphate (PIP3), the tail by the PI3-phosphatase PTEN that hydrolyses PIP3 to PI(4,5)bis-phosphate. To study de-novo cell polarization, we first depolymerized actin and subsequently recorded the spontaneous reorganization of actin patterns in relation to PTEN.

**Results:**

In a transient stage of recovery from depolymerization, symmetric actin patterns alternate periodically with asymmetric ones. The switches to asymmetry coincide with the unilateral membrane-binding of PTEN. The modes of state transitions in the actin and PTEN systems differ. Transitions in the actin system propagate as waves that are initiated at single sites by the amplification of spontaneous fluctuations. In PTEN-null cells, these waves still propagate with normal speed but loose their regular periodicity. Membrane-binding of PTEN is induced at the border of a coherent PTEN-rich area in the form of expanding and regressing gradients.

**Conclusions:**

The state transitions in actin organization and the reversible transition from cytoplasmic to membrane-bound PTEN are synchronized but their patterns differ. The transitions in actin organization are independent of PTEN, but when PTEN is present, they are coupled to periodic changes in the membrane-binding of this PIP3-degrading phosphatase. The PTEN oscillations are related to motility patterns of chemotaxing cells.

## Background

Patterns formed in the actin system of the cell cortex are the basis of cell motility, chemotaxis, cytokinesis, and phagocytosis. Subsets of actin-binding proteins determine the structure of actin assemblies, their anchorage to membranes, and the dynamics of their reorganization. Rapid polymerization and depolymerization of actin enable a cell to change its shape and local activities within seconds. Actin organization is regulated by signals from the environment, some of which are transmitted by soluble agents such as chemoattractants. However, the actin system also has a high capacity for self-organization, resulting in spatio-temporal patterns of actin structure and activity in the cell cortex.

In a variety of motile cells, shape changes have turned out not to be random. A pattern common to *Dictyostelium *cells [1], mouse embryonic fibroblasts, T cells, and wing disk cells [2] is the lateral propagation of protrusion and retraction waves along the membrane. In epithelial PtK1 cells, transversal wave formation is known to be controlled by Rac1 and its effector PAK [3]. In *Dictyostelium *cells these and other patterns have been shown to depend on the activities of PI3-kinases producing phosphatidyl-inositol (3,4,5)-tris-phosphate (PIP3) and on the PIP3 phosphatase PTEN [4]. Recently, the formation of new pseudopodia by alternating left-right splitting of existing ones has attracted attention, since it governs the orientation of cells in shallow gradients of chemoattractant [5] as well as unbiased cell motility [6]. These intrinsic spatio-temporal patterns are the outcome of non-linear interactions in the systems that control cytoskeletal activities.

Front and tail regions of a migrating cell are distinguished by proteins that determine the organization of filamentous actin in conjunction with the phosphoinositide pattern in the plasma membrane [7]. The Arp2/3 complex responsible for the nucleation of branched actin filaments is enriched at the front of the cell, and myosin-II, a motor protein that mediates retraction, is recruited to the tail. A positive signal for actin polymerization is provided by the activation of Ras at the front. Ras is proposed to act in a positive feedback circuit together with the membrane-bound lipid PIP3, which is also localized to the front [8]. In the highly motile cells of *Dictyostelium*, the actin and phosphoinositide patterns can be altered within seconds, thereby reprogramming the polarity of a cell. Here we study autonomous pattern formation in the actin system of *Dictyostelium *cells, using fluorescent markers for polymerized actin and for PTEN, a marker for the tail region of migrating cells.

**Figure 1:**
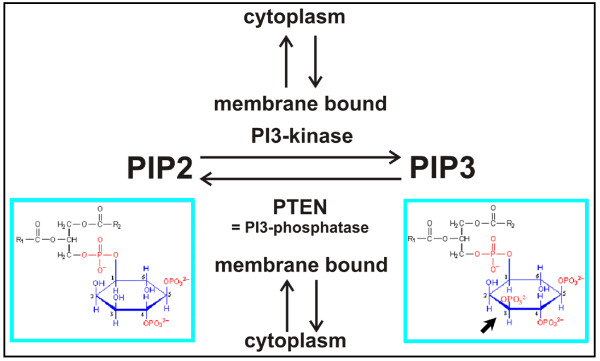
**Interconversion of the phosphoinositides PI(3,4,5)P3 and PI(4,5)P2 by phosphorylation/dephosphorylation at the 3 position of the inositol ring (bold arrow)**. In cells of *D. discoideum *both the PI3-kinases and the PI3-phosphatase PTEN shuttle between a cytoplasmic and an activated membrane-bound state. R1 and R2, hydrophobic membrane anchors of the phosphoinositides.

PTEN, a 3-phosphatase that inactivates PIP3 by its conversion to PIP2 (phosphatidyl-inositol (4,5)-bis-phosphate) plays an important role in the regulation of PIP3 (Figure 1). PTEN is stored in an inactive form in the cytoplasm; a small fraction of the total PTEN is reversibly bound in an active form to the plasma membrane [9]. PTEN contains multiple domains responsible for membrane binding, an essential one being an N-terminal PIP2 binding domain [10]. Consequently, binding to the membrane is up-regulated by PIP2, the product of PTEN activity, establishing a positive feedback circuit. PTEN binding to the membrane is negatively regulated by serine/threonine phosphorylation at the C-terminal tail [11]. The regulation of membrane binding is important for the responses to chemoattractant: in cells uniformly stimulated with cAMP, PTEN is released from the membrane; in cells that chemotax in a gradient of cAMP, PTEN remains bound to the membrane only at the lateral and posterior regions, as shown using GFP-PTEN [12]. This fusion protein is not only capable of membrane-binding but is also functional as a PI3-phosphatase that hydrolyzes PIP3 [9].

**Figure 2:**
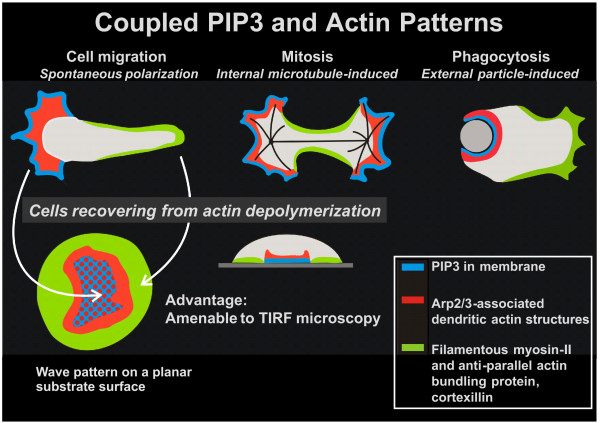
**Differentiation of the actin system in cell motility, mitosis, and particle uptake (top row), as compared to the wave pattern formed during a transient stage of recovery from actin depolymerization (bottom left)**. In this pattern actin waves surround an inner territory corresponding to the front of a motile cell and separate this area from an external one that corresponds to the tail of the cell. We are making use of the opportunity to apply TIRF microscopy on a planar substrate surface to visualize transitions between the two states of actin organization that are separated by a wave (bottom middle).

To explore the role of PTEN in symmetry breaking in the actin system, we monitored cells during reorganization of the cortical network of actin structures following the depolymerization of actin. When cells are treated with latrunculin A, an inhibitor of actin polymerization, the cells round up and become immobile. After removal of the inhibitor, the cells start within about 15 minutes to form circular actin waves that sharply separate two states of actin organization from each other, which correspond to actin organization at the front and tail, respectively, of a motile cell (Figure 2). In the inner territory enclosed by a wave, the plasma membrane is occupied by PIP3 [13]. The cortical region within this territory is dominated by the Arp2/3 complex, which is known to nucleate branched actin structures [14], while the external area is enriched in myosin-II and cortexillin, two proteins that bundle actin filaments in an anti-parallel manner [15]. Cortexillin is an actin-bundling protein that preferentially forms heterodimers of its two isoforms, cortexillin I and II [16].

Since the actin waves propagate along the planar surface of substrate-attached cells, the coupling of state transitions in the plasma membrane and actin cortex can be monitored using TIRF microscopy (Figure 2). This technique high-lights structures close to the substrate-attached cell surface, enabling us to monitor at sub-second resolution the correlation between the localization of PTEN and the conversion of actin structures.

Actin waves can propagate in one or the other direction, leading to either expansion or shrinkage of the inner territory. Thus, the actin waves are sites of state transitions in actin organization, which are correlated with the synthesis or hydrolysis of PIP3 in the underlying plasma membrane. PIP3 is generated at the site of the wave when the inner territory expands, and is degraded when the wave propagates in the opposite direction [13]. Moreover, the formation of actin waves is reversibly suppressed by the PI3-kinase inhibitor Ly-294002 [17]. The state transitions in the actin system may occur in a regular spatio-temporal pattern, the inner territory circulating with a period of about 5 minutes on the substrate-attached cell surface [15]. Accordingly PTEN, a constituent of the external area, tends to circulate as a crescent around the perimeter of the cells; importantly it does so even in the presence of 5 μM latrunculin A, suggesting an actin independent oscillator [18].

We show that during a transient stage of actin organization, the actin system periodically switches between a symmetric and asymmetric configuration. The switch to asymmetry is linked to the periodic pattern of PTEN-binding to the membrane. Nevertheless, state transitions in actin occur also in the absence of PTEN by the local initiation of propagating actin waves. With respect to the role of PTEN in symmetry-breaking, it is relevant that membrane-binding of PTEN is consistently induced at, and progresses from, the border of a PTEN-occupied membrane area.

## Results

### Protein and phosphoinositide patterns associated with actin waves

**Figure 3:**
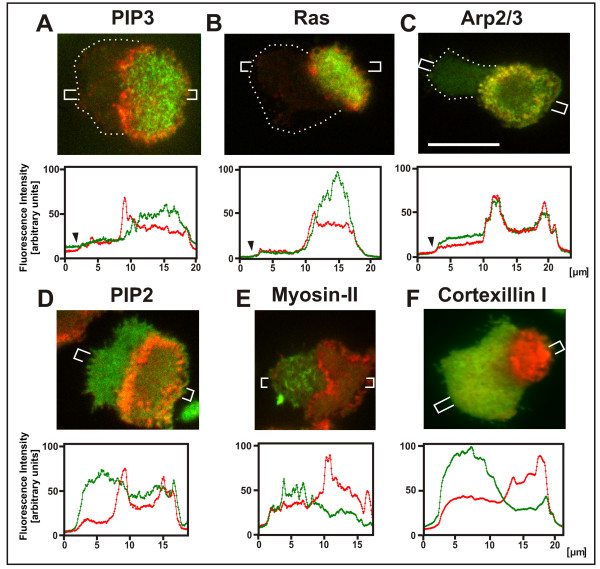
**Characteristics of the two territories separated by actin waves on the substrate-attached cell surface**. The actin waves labeled with mRFP-LimEΔ are shown in red, actin regulatory proteins or phosphoinositides in green. The upper panels depict TIRF images of double-labeled cells, the lower panels show line scans that cross the border between inner territory and external area. Sizes and positions of the scans are shown in the upper panels. **A**, the presence of PIP3 distinguishes the inner territory from the external one. **B**, activated Ras, a stimulator of actin polymerization, is bound to the membrane within the inner territory. This cell is also shown in Additional file 1 to ensure that the external area depleted of activated Ras is not out of focus. **C**, the Arp2/3 complex, responsible for the nucleation of branched actin assemblies, is enriched in the inner territory and in the actin waves surrounding this area. **D**, PI (4,5)-bis-phosphate (PIP2) is increased in the external area relative to the inner territory. **E**, filaments of myosin-II are localized to the external area. **F**, in this area also cortexillin is enriched, a bundling protein with a preference for the anti-parallel arrangement of actin filaments [16]. To prevent binding to PIP2, we probed for the actin structure using a truncated fragment (aa 352-435), which comprises the actin bundling domain without its C-terminal PIP2-binding motif [19]. Dotted lines in the images of (A to C) and arrowheads in the corresponding line scans indicate the cell border where otherwise hard to recognize. Scale bar for all images, 10 μ m.

Figure 3 presents an overview of components that are either enriched in the inner territory circumscribed by an actin wave (Figures 3A to 3C) or in the external area (Figures 3D to 3F). To enhance the production of circular actin waves on the substrate-attached surface of *Dictyostelium *cells, actin was depolymerized with latrunculin A and the drug then rinsed away. As actin polymerization recovered, the distribution of phosphoinositides and of proteins associated with different actin structures was monitored.

The localization of PIP3 sharply distinguishes the inner territory from the external one [13]; in the external area, this phosphoinositide is almost undetectable (Figure 3A). Similarly, Ras is strongly activated in the inner territory (Figure 3B and Additional file 1). The Arp2/3 complex, which primes the branching of actin filaments, is enriched in the inner territory and even more strongly within the actin waves (Figure 3C). In contrast, two proteins that favor anti-parallel assemblage of actin filaments, filamentous myosin-II and cortexillin [19], are substantially accumulated in the external area [15] (Figures 3E and 3F). PI(4,5)P2, a product of PIP3 dephosphorylation, proved to be weakly but consistently enriched in the external area (Figure 3D). The degree of this enrichment varied with the direction of wave propagation, as revealed by the fluorescence intensity of PLCδ1- GFP, a marker for PIP2 [20]. Relative to the fluorescence intensity of this marker in the inner territory, its intensity in the external area was 1.29-fold (s.d. ± 0.03) higher in front of an expanding wave and 1.72-fold (s.d. ± 0.21) higher behind a retracting wave (see also Additional file 2 and 3 for an expanding and a retracting wave, respectively).

These patterns in wave-forming cells display a differentiation of plasma membrane and cortical actin structures similar to that observed in motile cells, with the inner territory corresponding to the front region and the external area to the tail of a polarized cell. For experimental analysis, the actin wave patterns have the advantage of a much sharper separation of the territories than in the front - tail differentiation of a motile cell.

### PTEN pattern associated with expanding and retracting actin waves

**Figure 4:**
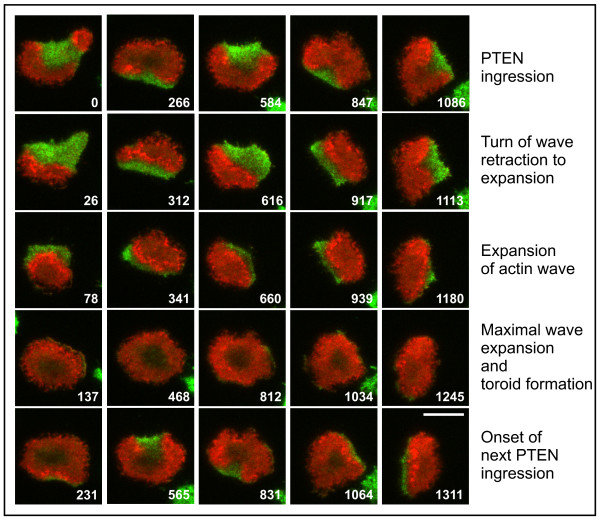
**PTEN dynamics (green) linked to the propagation of actin waves (red) in a cell recorded over a period of 21 minutes**. Within this time the cell undergoes five cycles of wave expansion and retraction. Selected frames of the time series are arranged such that the PTEN entry into the substrate-attached area is aligned in the top row of each period. Consecutive frames of a full period of retraction and expansion of actin waves are ordered in vertical columns below the respective frames of the top row. This arrangement shows that the starting site of each PTEN entry is located roughly opposite to the previous one. Within each period, the inner territory circumscribed by an actin wave shrinks to a minimal size and expands thereafter until it covers the entire substrate-attached membrane. Before PTEN ingresses again, the actin distribution assumes a toroid-like shape by the decline of polymerized actin in the central region. Time is indicated in seconds. Bar, 10 μ m.

In Figure 4 the periodic switches between symmetric and asymmetric actin patterns are related to the dynamics of membrane-bound PTEN (see also Additional file 4). There are two important features that this sequence displays. First, in the stage of maximal expansion of the actin wave, the central area of the substrate-attached cell surface is largely depleted of actin, creating a toroid-like appearance. This depletion is not due to an increase in membrane-bound PTEN. Second, transition to asymmetry begins with the unilateral decrease in filamentous actin, generating a horseshoe shape with an opening where PTEN ingresses from the perimeter of the cell. The sites of PTEN-ingression alternate, as revealed by the images aligned in the top row of Figure 4: PTEN displaces the actin-rich area alternately from the top or the bottom of the frame.

### Actin waves in PTEN-null cells

**Figure 5:**
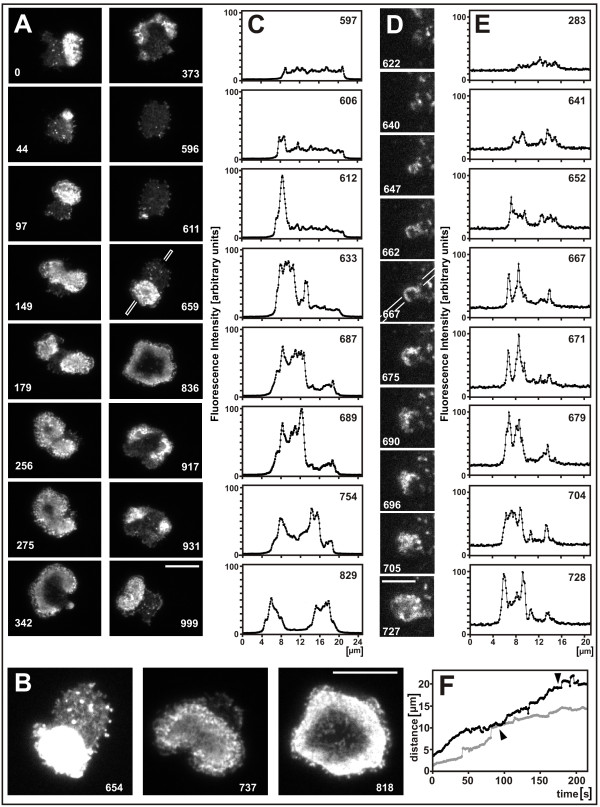
**Actin waves in PTEN-null cells expressing LimEΔ-GFP to label actin filaments**. The mutant cells were pre-treated with 5 μM latrunculin A similar to GFP-PTEN expressing cells. **A**, characteristic actin patterns selected from a continuous recording of a single cell. **B**, actin network structures. Left panel: increased brightness renders the loose network in front of the wave visible, but the area circumscribed by the wave is overexposed. Middle: at lower brightness, the dense network in the inner territory and clusters within the wave zone are resolved. Right: toroid-like stage showing disruption of the actin network in the central area. **C**, initiation and propagation of a wave front scanned along the line indicated in the 659 s frame of (A). **D**, the first actin wave initiated in another cell that recovers from treatment with latrunculin A. **E**, line scan through the site of wave initiation as indicated in the 667 s frame of (D). Numbers in (A) to (C) indicate seconds after the beginning of Additional file 5, numbers in (D) and (E) of Additional file 6. **F**, speed of propagation of the wave fronts along the scans in C, (black curve) and E, (gray curve). Arrowheads indicate arrival of the wave front at the cell perimeter; subsequently wave propagation causes slow expansion of the substrate-attached cell surface. Jumps in wave propagation are due to the deposition of new actin clusters at the wave front, as seen in the Movies. Bars, 10 μ m in (A) and 5 μ m in (D).

The link between actin and PTEN dynamics raised the question of whether PTEN is essential for the pattern of actin waves, in particular for the regular alternation of wave expansion and retraction. Figure 5A and Additional file 5 exemplify the repertoire of actin dynamics in PTEN-null cells during their recovery from actin depolymerization. Actin waves can be formed in the absence of PTEN; they expand, split into two, or fuse (Figure 5A, 97 - 275 s frames), as they do in wild-type cells [21]. The formation of a toroid-like structure is also a PTEN-independent phenomenon (836 s frame). The two territories separated by the actin waves have actin structures similar to those previously reported for wild-type cells [15]: the external area contains a loose network of bundled actin filaments (Figure 5B, left panel), whereas the inner territory is filled with a dense fabric of filamentous actin (Figure 5B, middle panel). In the toroid-like configuration formed at the end of wave expansion, the actin is enriched along the perimeter of the substrate-attached cell surface and becomes largely disassembled in the central region (Figure 5B, right panel), similar to GFP-PTEN-expressing cells (Figure 4).

However, the wave dynamics in PTEN-null cells is distinguished from that in wild-type cells by the absence of a regular alternation of wave expansion and retraction. Although the waves become fragmented and sometimes completely extinguished in the mutant cells (Figure 5A, 373, 596, and 917 s frames), their retraction does not occur in the form of a circular wave surrounding an inner territory, as it is typical of wild-type cells [15]; see also Figure 4 and Additional file 4 of the present paper.

### Actin fluctuations and the local switching on of state transitions

The PTEN-null cells enabled us to study the initiation and propagation of actin waves unaffected by any antagonistic activity of PTEN. The initiation of an actin wave can be subdivided into three phases (Figures 5A, C and 5D, E). In the first phase, only highly mobile clusters of variable shapes are recognizable. In the second phase, a circular structure of polymerized actin is stabilized, densely populated with actin filaments. In the third phase, this area expands until the state transition from external to inner area propagates in the form of an actin wave across the entire substrate-attached surface of the cell (Figure 5A, 596 - 836 s frames, and Additional files 5 and 6).

Details of the initiation of an actin wave are illustrated in Figure 5D. Among the earliest structures formed during recovery from actin depolymerization are small clusters of polymerized actin. In wild-type cells we have shown that the majority of these clusters are associated with clathrin and involved in endocytosis [15]. In addition, polymerized actin structures of various shapes are transiently formed, including propagating wave fronts with open ends. Only rarely do these fluctuations result in the initiation of a circular wave, the critical step being the formation of an imperfect ring of actin (Figure 5D, 662 and 667 s frames), which is subsequently filled with short-lived, dense clusters of actin.

Line scans across the initiation sites display patterns of fluorescence intensities in quantitative terms. The scans of Figure 5C comprise stages of the wave depicted in Figure 5A, from the initiation (606 to 633 s frames) up to a late stage of propagation (829 s frame). During the onset of propagation, a compact area of high fluorescence intensity is split, in the scan direction, into two flanking wave fronts (frames 687 and 689). Similarly, the line scans in Figure 5E, taken from the images of Figure 5D, show an initial actin ring (667 and 671 s frames) and the filling-up of the space in between (679 and 704 s frames), before the wave starts to propagate (728 s frame).

Once initiated, an actin wave is capable of propagating across the entire substrate-attached area with an average velocity of 6.5 μ m per minute. There are phases of faster or slower propagation, but the velocity does not systematically diminish with increasing distance from the site of initiation (Figure 5F). This means, a stimulus for transition from the state of the external area to that of the inner territory is continuously renewed at the wave front, analogous to the progression of a bush fire.

### PTEN localized to the non-attached membrane area

**Figure 6:**
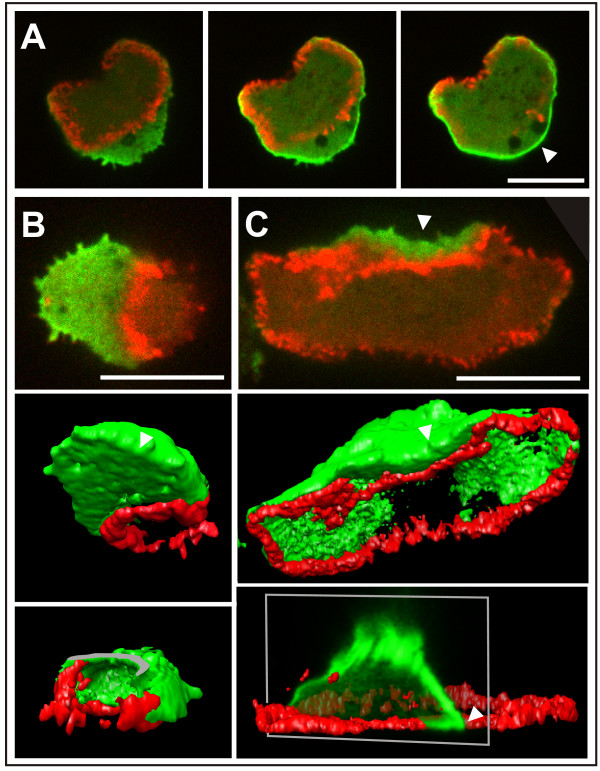
**Three-dimensional distribution of PTEN (green) in relation to actin waves (red)**. Wave-forming cells expressing GFP-PTEN and mRFP-LimEΔ were scanned by spinning-disc confocal microscopy. **A**, three optical sections from a z-stack scanned at the substrate (left), and 600 nm (middle), or 1200 nm ahead of the substrate (right). This series indicates that PTEN in the external area of the substrate-attached surface is contiguous with PTEN bound to the membrane of the non-attached surface (arrowhead). **B**, three-dimensional PTEN pattern in relation to an actin wave. The reconstruction shows the continuity of the PTEN-rich area at the border of the free and substrate-attached cell surface (arrowhead in the middle panel) and membrane location of the PTEN (bottom panel). To view the inside of the cell, its top has been optically cut off in the bottom panel, and the plane of the cut-off section shown in gray. **C**, 3D-reconstruction similar to (B). The PTEN-rich border between free and substrate-attached cell surface is seen (arrowheads). A cross-section through the stacks of PTEN images (white frame in the bottom panel) shows the corner of the PTEN-rich area in a side view. Bars, 10 μ m.

The finding that PTEN enters the substrate-attached area of the plasma membrane always from the cell perimeter suggested that PTEN incursions seen by TIRF microscopy are in fact extensions of the non-attached cell surface into the substrate-attached area. The 3-dimensional patterns of PTEN reconstructed from stacks of confocal sections confirmed this notion. They indicate a coherent membrane area occupied with PTEN on the free surface of a cell, from which PTEN indentates into the substrate-attached area up to the outer rim of an actin wave (Figure 6 and Additional files 7, 8 and 9).

**Figure 7:**
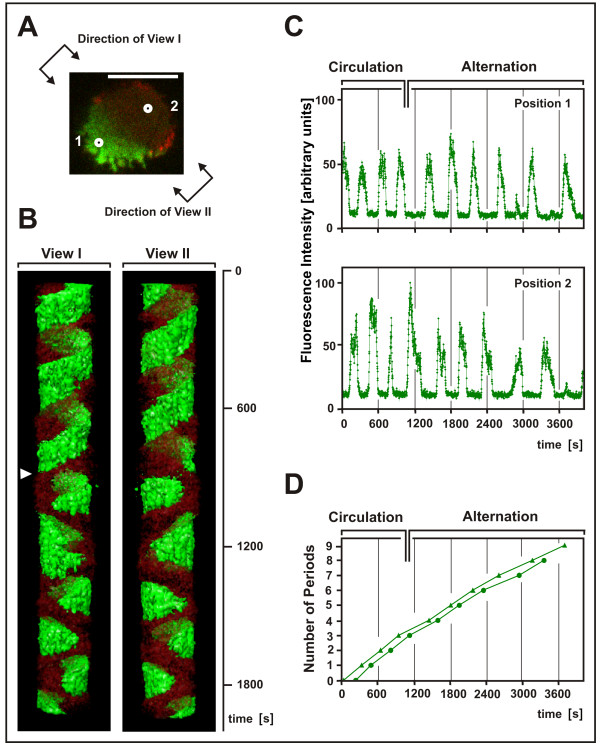
**Change of spatiotemporal PTEN pattern from circulation to alternation**. A cell expressing GFP-PTEN (green) and mRFP-LimEΔ (red) has been incubated with 2 μM latrunculin A and recorded for more than one hour. PTEN started to circulate, but subsequently turned into an alternating mode of lateral ingression into the substrate-attached area of the cell surface. **A**, image at the beginning of recording, depicting the crescent of PTEN. The directions of views I and II in (B) are shown, and the positions of scans 1 and 2 in (C) are indicated as white circles. Bar, 10 μ m. **B**, temporal pattern evolution. The spatial pattern is reconstructed along the time axis from top to bottom. The first part of the recording is shown, comprising the turn from circulation to alternation (arrowhead). Contributions of fluorescence from an adjacent cell have been eliminated in this graph. (The border of this second cell is seen in Additional file 10). **C**, periodic peaks of the GFP-PTEN fluorescence at the two positions indicated in (A). Since these positions differ by 180°, phases of the oscillations are accordingly shifted. **D**, number of completed periods plotted over time determined at position 1 (triangles) or position 2 (circles). The slope of the curves does not indicate an increase in frequency. It rather suggests a slight decrease after the turn from circulation to alternation. (The small peaks seen in (C) at 2894 and 3720 s have been disregarded).

To uncouple the dynamics of PTEN from the formation of actin waves, cells were incubated with 2 μM latrunculin A, a critical concentration for pattern formation in the actin system: in a fraction of cells no actin waves were detectable, while in other cells rudimentary waves were observed. Under these conditions, PTEN still periodically entered the substrate-attached membrane area from its perimeter as previously shown by Arai et al. [18], often in the form of crescents that circulated with an average period of 5.8 minutes around the cell border (Figure 7A, B). Dark spots in the PTEN layer, most likely clathrin-coated pits, served as stationary markers, verifying that the pattern of membrane-bound PTEN rotated, while the cell as a whole did not (data not shown).

The temporal patterns measured at single points close to the perimeter of the cell revealed non-sinusoidal oscillations that displayed two states of the membrane, PTEN-occupied or PTEN-depleted, with sharp on and off switches between the two states (Figure 7C). The PTEN peaks proved to be differently structured: a phase of increase may turn with no delay into a decrease, or an interval of fluctuations at a high level may separate the rise and fall of PTEN. The decay of PTEN can occur stepwise, with arrests at one or two levels between the peak and baseline. The average time required for transition from the PTEN-depleted to the PTEN-rich state of the membrane was 60 s (cases with arrests at intermediate plateaus not considered), the time for the reverse transition was 73 s. Consistently, the PTEN peaks were separated by extended phases with remarkably small fluctuations in their low fluorescence intensities. Meanwhile the actin label displayed no detectable oscillations, although dynamic clustering was still observed (Additional file 10).

Circulation of PTEN can convert, without any change in the external conditions, into a pattern of periodic ingression from alternating sides of the cell perimeter (Figure 7B, C and Additional file 10). This conversion may occur in one step by cessation of the PTEN circulation and commencement of the alternating PTEN ingression. In Figure 7B the time of conversion is indicated by an arrowhead. To examine whether the conversion of the spatial pattern has any effect on the period of the oscillations, we determined the interval between PTEN peaks before and after the turn from circulation to alternate ingression. Measured at two opposed positions of the substrate-attached cell surface as indicated in Figure 7A, only a minor phase shift was associated with conversion of the pattern; nor did the frequency of the PTEN oscillations increase (Figure 7D). A characteristic of the alternating PTEN patterns is that, with a few exceptions, the areas of PTEN recruitment expanding from one side or the other did not overlap. As a result, the PTEN peaks at positions 1 and 2 were separated from each other not only in time but also in space. Exceptions were the small peaks at 2894 s in position 1 and at 3720 s in position 2, which indicate a tendency to frequency doubling because of a slight overlap of the areas.

### Dynamic membrane-binding of PTEN

**Figure 8:**
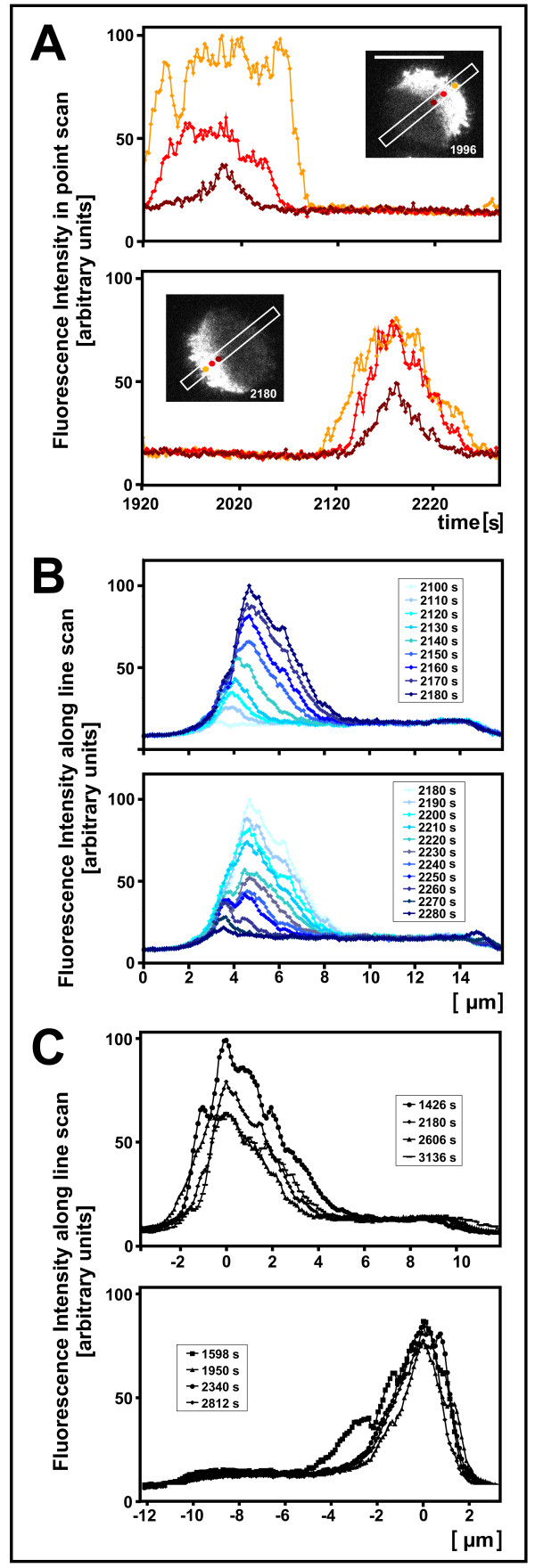
**Spatial and temporal profiles of rising and falling PTEN gradients**. Temporal (A) and spatial (B, C) patterns of GFP-PTEN during alternating crescent formation are quantitatively analyzed. **A**, time course of fluorescence intensities during two half-periods shown in the upper or lower panel, respectively. In each panel, PTEN dynamics is plotted for three positions at different distances from the cell perimeter as indicated by color-coding in the insets. These insets show the fully developed crescents of PTEN in the two half-periods and indicate the positions of line scans used for (B). **B**, spatial PTEN patterns during generation (upper panel) and decline of a gradient (lower panel). The color-coding refers to time in seconds as indicated in the insets. **C**, compilation of the shapes of eight fully developed PTEN gradients. Gradients of four half-periods ingressing from the left side are shown in the upper panel, gradients of the four alternating half-periods ingressing from the right side are shown in the lower panel. The curves are aligned by setting the maxima of fluorescence intensities to zero on the space coordinate. These data are based on the recording shown in Additional file 10; times in (A) and numbers in the insets of (B) and (C) indicate seconds after beginning of the movie. These numbers correspond also to the scale of seconds in Figure 7. Images have been acquired at frame-to-frame intervals of 2 seconds. For (B) and (C), data from 5 frames have been averaged.

The alternation between a PTEN-rich and PTEN-depleted state in a defined spatio-temporal pattern offers a possibility to analyze the mode of state transitions using TIRF microscopy. Transition to the PTEN-rich state began at the cell perimeter and propagated from there into the substrate-attached area (Figure 8A). During all stages of increasing and decreasing PTEN-binding to the membrane, the PTEN-gradients maintained their maximum at the cell perimeter (Figure 8B). At the state of maximal extension, the PTEN gradients declined straight toward the center of the substrate-attached area, with a low, uniform background of PTEN in the other half of the area (which might primarily reflect unbound PTEN) (Figure 8C). These data indicate that a signal enhancing PTEN binding is generated in an oscillating manner at the border of the PTEN-rich unattached area of the membrane, allowing the PTEN-binding capacity to extend as a crescent into the substrate-attached area. The shape and limited expansion of the gradients suggest a counteracting factor that periodically prevents binding of PTEN to, or favors its dissociation from, other regions of the substrate-attached membrane.

**Figure 9:**
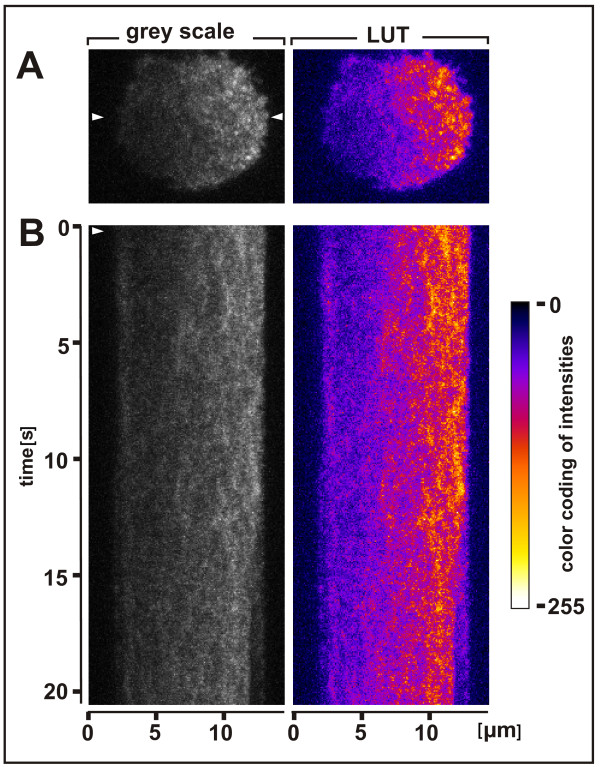
**Short-term structures formed by membrane-bound PTEN**. A cell treated as the one shown in Figure 7, was recorded at 10-millisecond intervals. **A**, PTEN-rich territories became most distinct in average projections of five frames. **B**, in the kymograph of a slice through the PTEN crescent, short-term persistence of these territories is displayed by vertical stripes. Average projections of five frames recorded at 10-millisecond intervals. The left panels show 8-bit gray scale images. In the right panels the fluorescence intensities are color-coded using a "fire" lookup table.

The formation of sharp PTEN patterns is hardly conceivable with a free diffusion of single PTEN molecules in the membrane. To visualize short-lived structures in the PTEN gradients, we have recorded PTEN patterns at a frequency of 100 Hz. GFP-PTEN turned out to cluster in the membrane into rapidly changing patterns (Figure 9 and Additional file 11). Average projection of stacks of frames revealed areas of preferred PTEN localization with a persistence in the order of 50 ms. It appears therefore that there is a scaffold that causes PTEN, in spite of its high mobility, to reside preferentially within micrometer-sized domains of the membrane. Since under the conditions used actin is not completely depolymerized, the scaffold might be made of a loose network of actin filaments.

## Discussion

### Coupling of actin and PTEN dynamics

The rationale of the experimental study presented here is to abrogate polarity in the cell cortex by the depolymerization of actin, and to monitor the emergence of asymmetry during reorganization of the actin system. The basic result is that actin reorganization involves a period of repeated events of symmetry breaking before normal front-to-tail polarity and cell motility are regained. In this transitory period of fluctuating polarization, the dynamics of pattern formation can be considered as a combination of two periodic processes. One is the PIP3-controlled patterning of the actin system, the other is the lateral ingression of the PIP3-degrading enzyme PTEN. These patterns are of interest as examples of self-organization; they generate intracellular compartments without a need for membranes to separate them.

The actin dynamics in the transition stage of recovery from actin depolymerization is characterized by the formation of circular waves at the substrate-attached cell surface. These actin waves enclose an inner territory that differs from the external area in the high PIP3 content of the membrane and in the actin organization of the cell cortex (Figure 3). When an actin wave arrives at the cell perimeter, the substrate-attached cell surface is in a symmetric state. The crucial event in symmetry breaking is the recruitment of PTEN to one side of the substrate-attached membrane area in combination with the lateral opening of the actin wave, creating a horseshoe-like pattern (Figure 4). These data imply that asymmetry in the actin pattern is generated during transition from the state of the inner territory to that of the external area, which becomes occupied by PTEN.

Both the control circuits of PTEN and of the actin network in the cell cortex undergo reversible transitions between two states. PTEN oscillates between a state of high and a state of low membrane binding. The actin system alternates between one state dominated by the Arp2/3 complex and another state characterized by high affinity for filamentous myosin-II and cortexillin, a protein that interacts with anti-parallel bundles of actin filaments [15].

The actin and PTEN patterns are linked to each other by mutual exclusion. However, these patterns are not strictly complementary: in the development of a toroid-like pattern, actin declines without an increase in PTEN (Figure 4, 137 s frame and corresponding frames in subsequent periods). This decline is associated with the down-regulation of PIP3 [13]. Together, these data indicate that net depolymerization of actin is caused by two mechanisms, a PTEN dependent and an independent one.

The dynamics of actin and PTEN patterns requires non-linear interactions in the control circuits of the pattern forming elements. A positive feedback circuit for the membrane-binding of PTEN has been postulated by Iijima *et al*. [10]: the N-terminal domain of PTEN comprises a PIP2 binding site, implying that the product of PTEN activity enhances the binding and consequently the activity of PTEN in a membrane area [11]. According to this view, the PIP2 density in the membrane of the external area, which becomes occupied by PTEN, should be higher than in the membrane of the PTEN-depleted inner territory. Indeed, the PIP2-recognizing PH domain of human PLCδ1 indicated an increase in PIP2 in the external area relative to the inner territory. However, the PIP2 ratio was less than 2, which would require a high cooperativity of PTEN interaction with PIP2 in order to cope with the strong difference in PTEN occupancy between the two areas. Moreover, the distribution of the PIP2-label does not coincide with that of PTEN: wheras the PIP2-label indicates a sharp increase in front of an expanding actin wave, PTEN forms a gradient with a peak at the perimeter of the substrate-attached area.

PH-PLCδ1 binds also to the degradation product of PIP2, I(1,4,5)P3. Therefore, the possibility should be taken into account that this compound influences the PIP2 assay [22]. However, since IP3 is soluble, we would not recognize it in TIRF. The remaining possibility that PLCδ1 is depleted by a high local concentration of IP3 in the cytoplasm is unreasonable since diffusion through the small cells of *Dictyostelium *is fast and would not allow to create a spatial pattern: the diffusion coefficient for GFP in the cytoplasm is 24 μ m^2 ^× s^-1 ^[23].

Factors other than PIP2 will contribute to the membrane binding of PTEN. An additional factor is probably the regulation of PTEN phosphorylation by membrane-bound phosphatase and/or kinase. The strong membrane binding of unphosphorylatable PTEN [24] suggests that a membrane area that is populated by a serine-threonine phosphatase would convert cytosolic PTEN to a membrane-bound state.

A positive feedback circuit for PIP3-coupled actin polymerization involving Ras activation has been proposed by Charest and Firtel [25] and Sasaki *et al*. [8]. The activity of PI3-kinases 1 and 2 of *D. discoideum *depends on their Ras-binding domains [12]. An antagonistic interaction between PTEN and actin is given by the PI3-phosphatase activity of PTEN, which degrades PIP3 and thus terminates its stimulation of actin polymerization.

Circulation of an activating process, as the one inducing PTEN ingression around the perimeter of the cell, can be modeled assuming a reaction-diffusion system consisting of an activator and two inhibitors [26]. According to this model, the activator is formed by an autocatalytic reaction. A long-range inhibitor with a short time constant is responsible for the patterning in space and a short-range inhibitor with a long time constant for the patterning in time. The second inhibitor may be replaced by a slow deactivation process or by the depletion of a factor required for activation. A reaction-diffusion model specifically based on the reciprocal negative relation of membrane-bound PTEN and PIP3 has been shown by Arai et al. [18] to simulate periodic wave formation in the phosphoinositide system.

### Initiation and propagation of actin waves in the absence of PTEN

The question of whether state transitions in the actin system depend on the dynamics of PTEN has been addressed by recording actin patterns in PTEN-null cells. In these mutant cells, the onset of actin polymerization could be studied without any antagonistic effect of PTEN. Actin waves originated as a rare event from fluctuations in actin polymerization when a patch about 2 μ m in diameter populated with a dense network of actin filaments became stabilized. From this initiation site, actin waves started to propagate, thus converting progressively a loose network of actin filaments into a dense fabric (Figures 5A, B). As previously shown for wild-type cells, this state transition is associated with the replacement of two actin-bundling proteins, myosin-II and cortexillin, by the strong recruitment of the Arp2/3 complex [27,15]. Once initiated, an actin wave propagated in PTEN-null cells with an average velocity of 6.5 μ m per minute across the membrane (Figure 5F), in accord with the velocities previously reported for wild-type and SCAR-null cells [27]. The actin structure within the area surrounded by an expanding wave differed in its dense filament arrangement from the loose network in the external area (Figure 5B), similar as in wild-type cells.

These findings indicate that PTEN is not required for state transitions in the actin system and also not for the propagation of actin waves, although it appears to be important for the regular periodicity of state transitions. The question of an inherent bistability in the actin system of the cell cortex has been addressed by Beta [28], who explored conditions under which the actin system may switch between two states of different structure.

### Actin and PTEN dynamics are based on different modes of state transitions

**Figure 10:**
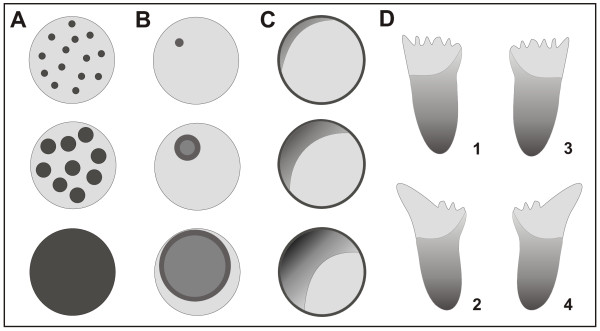
**Modes of state transitions in the actin-phosphoinositide system of the cell cortex**. **A**, growth and coalescence of patches. This mechanism has been proposed by Gamba *et al*. [29] for the accumulation of PIP3 at the front of chemotaxing cells. (Adopted from [30]). **B**, state transition initiated as a rare event in a field of damped fluctuations. Once initiated, the state transition progresses as a wave, as demonstrated for the transition of actin organization (Figure 5). **C**, extension of a gradient linked to the border of a persisting area. This mode of expansion of a coherent area applies to the formation of PTEN gradients (Figure 6). **D**, transformation of the pattern in (C) to proposed PTEN oscillations in a chemotaxing cell. The border of the PTEN-rich membrane area (dark) is supposed to be asymmetric and to suppress actin polymerization and pseudopod protrusion alternately at the right (1, 2) or left (3, 4) side of the front region.

The study of symmetry breaking in the actin system revealed different modes of state transitions that determine the type of patterns generated. At least three possibilities can be distinguished of how transitions from a state 1 to state 2 are initiated in a bistable system (Figure 10). (1) As proposed by Gamba *et al*. [29,30] for the generation of PIP3 patterns in chemotaxis, fluctuations in the area of state 1 may be amplified at multiple sites to form patches of state 2. Eventually, growth and coalescence of these patches results in conversion of a coherent area from state 1 to state 2 (Figure 10A). (2) Local initiation of a transition from state 1 to state 2 may be a rare event but, once initiated at a single site, conversion to state 2 will propagate across the entire area of state 1 (Figure 10B). This mode of state transition is exemplified by the actin waves shown in Figure 5 and Additional files 5 and 6. Autocatalytic transitions that propagate in the form of a wave have been modeled on the basis of an array of kinase molecules undergoing intermolecular autophosphorylation [31]. This mechanism has been shown to apply to the lateral propagation of EGF receptor phosphorylation at the plasma membrane [32]. (3) State 2 may persist in a coherent area, at the border of which the transition of state 1 to state 2 is induced (Figure 10C). This mode of state transition holds for the gradients formed during the lateral ingression of PTEN onto the substrate-attached membrane (Figures 6, 8B and 8C). The inner territory circumscribed by an actin wave appears as a hole in the coherent membrane area occupied by PTEN. The inner territory corresponds to the front region of a motile, polarized cell and accordingly to PIP3 patches at the front of a cell stimulated by chemoattractant [33].

### Biological relevance of coupled PTEN and actin patterns

The separation of actin structures in a wave-forming cell resembles the front-tail differentiation in a motile cell [15]. The actin-rich area occupied by the inner territory and the wave itself corresponds to the front region of a cell, and the external area to its tail: the front is rich in the Arp2/3 complex and in PIP3, the tail in filamentous myosin-II responsible for retraction (Figure 3). A similar differentiation is observed in cytokinesis when the cleavage furrow is enriched in cortexillin and myosin-II, but depleted of the Arp2/3 complex. Furthermore, the actin wave pattern resembles closely actin organization in phagocytosis, the inner area corresponding to the PIP3-rich membrane of a phagocytic cup induced by the attachment of a particle, and the actin wave conforming to the rim of the cup [13].

A cell migrating in a shallow gradient of chemoattractant tends to protrude pseudopods alternately in directions right or left of the existing one [5]. Based on these data, a self-organized cycle has been proposed by Insall [34] to underlie pseudopod formation in chemotaxis. Split pseudopod formation is comparable to the actin and PTEN patterns in the wave-forming cells studied here (Figure 10D). In the symmetric toroid-like state, actin is accumulated in a ring, indicating that the boundary of the area rather than the center is the preferred site of actin polymerization (Figure 4). During lateral PTEN ingression, actin polymerization is asymmetrically inhibited, resulting in the alternating or circulating dominance of one or the other sector of the actin ring.

## Conclusions

During recovery of actin organization in the cell cortex after depolymerization, actin exists in a bistable state, and transitions between these states are marked by propagating waves. Periodicity of state transitions in the actin system is coupled to oscillatory membrane-binding of PTEN.

Nevertheless, actin can switch also in the absence of PTEN between two states that have similar characteristics as those formed in the presence of PTEN. State transitions in actin and PTEN are based on different principles. Changes in actin organization are initiated de-novo at single sites and propagate from there in the form of waves over a large territory, up to the entire substrate-attached cell surface. The membrane-binding of PTEN is induced at the border of a compact membrane area already occupied by PTEN. The expanding and retracting PTEN gradients at the border of this area are composed of domains of highly mobile and clustering PTEN molecules. In summary, patterns in the actin system are determined by the interconnection of two principles of state transitions.

## Methods

Cells were harvested from sub-confluent cultures with nutrient medium in plastic petri dishes, transferred to glass coverslips on which a plexiglass ring of 19 mm diameter was mounted using paraffin, and washed twice with 17 mM Na/K-phosphate buffer, pH 6.0 [27]. The cells were cultivated and imaged at 23 ± 2°C.

Cells expressing PTEN-GFP in a PTEN-null background [9] obtained from Peter Devreotes through the Dicty stock center (strain DBS0236831), were transfected to express mRFPm-LimEΔ [35] using 33 μg/ml of hygromycin for selection; clones employed were 274-2-6 and 274-4-10. PTEN-null cells of AX2 [36] obtained from Rob Kay through the Dicty stock center (strain DBS0252655), were transfected for LimEΔ-GFP expression using 10 μg/ml of G418; the clone employed was 293-1-2. Other strains used are compiled in Table 1.

**Table 1:** Previously published double-labeled strains

GFP label	mRFP label	Clone	Drug resistance	Parent strain	Reference
PLCδ1, PH domain	mRFP-LimEΔ	252-1-12	B10/G10	AX2-214	[38]
Raf1-RBD	mRFP-LimEΔ	278-2-10	B10/G10	AX2-214	[38]
Cortexillin I, actin-bundling fragment 352-435	mRFP-LimEΔ	165-2-5	B10/G10/H66	Cortexillin I null in AX2-214	[15]
Myosin-II heavy chain	mRFP-LimEΔ	75-2-2-11	B10/G10	AX2-214	[15]
Arp 3	mRFP-LimEΔ	1	B10/G10	AX3	[39]
PHcrac	mRFP-LimEΔ	230-1-1-2	B10/G10	AX2-214	[39]

B 10, B 20: 10 μg/ml, 20 μg/ml blasticidin G 10: 10 μg/ml G 418 H 66: 66 μg/ml hygromycin

### TIRF microscopy

Through-objective TIRF imaging was performed using an Olympus IX 71 microscope and an Andor iXon + camera as described previously [27]. The pixel size was 0.106 μm. The width of line scans was 16 pixels in Figures 3 and 8, 1 pixel in Figure 9, and 8 pixels in Figure 5. GFP was excited at 491 and mRFP at 561 nm. Both fluorophores were excited simultaneously and the emissions split. Both fluorophores were excited simultaneously and the emissions split using a Hamamatsu W-View image splitter (Semrock emission filters BL HC 525/30 and BL HC 617/73 for GFP and mRFP, respectively). The TIRF images were analyzed using Fiji (http://pacific.mpi-cbg.de/wiki/index.php/Fiji), an image processing package based on ImageJ (http://rsb.info.nih.gov/ij).

### Spinning disc microscopy

The 3-dimensional images were acquired by recording z-stacks at 200 nm distances using an Olympus/Andor spinning disc microscope (Avon, MA, USA) with a 60x PlanApoN oil objective, NA 1.42 [37]. Images acquired at 488 and 561 nm excitation were filtered through Semrock emission filters and recorded using an iXon+ EMCCD camera. For 3D-reconstructions, the images were processed using the alpha version 1.3 of UCSF Chimera developed by the Resource for Biocomputing, Visualization, and Informatics (http://www.cgl.ucsf.edu/chimera/).

## Authors' contributions

GG participated in experiments and structured the paper, ME did the spinning disc recordings and analyzed data, DW produced the transformants and BS-D was responsible for the TIRF recordings. All authors read and approved the final manuscript.

## Supplementary Material

Additional file 1 **Movie 1, Strict localization of activated Ras (green) to the inner territory**. This movie shows the same cell as Figure 3B. The left panel displays the actin label (red) at low brightness, appropriate to visualize the actin wave and inner territory. For the right panel, the actin label has been enhanced to visualize membrane-associated actin patches in the external area. As reported previously [15], most of these patches are linked to clathrin-dependent endocytosis. Click here for file

Additional file 2 **Movie 2, PIP2 dynamics associated with an expanding actin wave**. The cell expressed PH-PLCδ1-GFP as a label for PI(4,5)P2 and mRFP-LimEΔ for filamentous actin. During propagation of the wave, the PIP2 label declined while the external area in front of the propagating wave was converted into internal territory. The left panel shows fluorescence in the mRFP channel, the middle panel simultaneously recorded fluorescence in the GFP channel. The right panel displays fluorescence intensities of PH-PLCδ1-GFP (green) and mRFP-LimEΔ (red) along the line scan superimposed on the images in the first frame. Frame-to-frame interval 1 s. Click here for file

Additional file 3 **Movie 3, PIP2 dynamics associated with a retracting wave**. This is a continuation of Movie 2, showing increase of the PIP2 label in the external area behind the actin wave. The position of the line scan is adjusted according to the direction of wave propagation. Frame-to-frame interval 1 s. Click here for file

Additional file 4 **Movie 4, Symmetric and asymmetric actin patterns linked to lateral PTEN ingression**. The cell co-expresses GFP-PTEN with mRFP-LimEΔ as a label for filamentous actin. The cell is captured in the stage of actin-wave formation during recovery from actin depolymerization using 5 μM latrunculin A. This movie shows the same sequence as Figure 4. Left panel: merged fluorescence images displaying the PTEN label in green and the actin label in red. Middle panel: separate channel showing the fluorescence of GFP-PTEN. Right panel: separate channel showing the fluorescence of mRFP-LimEΔ. Frame-to-frame interval 1 s. Click here for file

Additional file 5 **Movie 5, Initiation and propagation of actin waves in a PTEN-null cell**. The cell expresses LimEΔ-GFP as a label for filamentous actin. The cell is recovering from treatment with 5 μM latrunculin A; this Movie shows the same recording as Figure 5 A - C. Frame-to-frame interval 1 s. Click here for file

Additional file 6 **Movie 6, Initiation and propagation of an actin wave in a PTEN-null cell similar to Movie 5**. This recording begins with a 10-minutes run of actin fluctuations before a wave is initiated. The movie shows the same recording as Figure 5D and E. Frame-to-frame interval 1 s. Click here for file

Additional file 7 **Movie 7, Three-dimensional reconstruction of a cell expressing GFP-PTEN (green) and mRFP-LimEΔ (red) as shown in Figure 6B**. This reconstruction focuses on the continuity of the PTEN-rich area of the substrate-attached cell membrane with the bulk area of the non-attached membrane. The inner territory circumscribed by the actin wave is depleted of PTEN. The cell has been optically unroofed (white plane). Click here for file

Additional file 8 **Movie 8, Reconstruction of a cell similarly labeled as the cell in Movie 7**. This cell is also shown in Figure 6C; it illustrates the extension of the PTEN-rich free surface into the substrate-attached area outside the actin wave. Click here for file

Additional file 9 **Movie 9, Segmented view of the cell shown in Movie 8**. The actin wave (red) is combined with a moving plane that displays the fluorescence distribution of GFP-PTEN (increasing intensities color-coded from dark-green to white). The plane illustrates the coherence of the PTEN-rich area on the substrate-attached membrane with the PTEN layer on the free cell surface. Since we did not manipulate the image by deconvolution, the top of the cell appears fuzzy. Click here for file

Additional file 10 **Movie 10, Turn from the circulation of PTEN to alternate ingression into the substrate-attached cell surface**. The cell was incubated with 2 μM latrunculin A. Left panel: merged fluorescence of GFP-PTEN (green) and mRFP-LimEΔ (red). Middle panel: separate channel showing the fluorescence of GFP-PTEN. Right panel: separate channel showing the fluorescence of mRFP-LimEΔ. Frame-to-frame interval 2 s. Click here for file

Additional file 11 **Movie 11, Dynamic clustering of membrane-bound PTEN**. This movie corresponds to Figure 9, showing PTEN patterns averaged over frame-to-frame intervals of approximately 50 ms. The left panel displays fluorescence intensities of GFP-PTEN on a 8-bit gray scale, the right panel the same data represented as a "fire" lookup table. PTEN clustering is most prominent within the crescent on the right of the cell. Click here for file
